# Automated bone property analysis using corrected in vivo dental cone-beam CT data of human wrists

**DOI:** 10.1038/s41598-024-75222-6

**Published:** 2024-12-16

**Authors:** Benjamin Klintström, Anna Spångeus, Alexandr Malusek, Alexander Synek, Mischa Woisetschläger, Dieter Pahr, Eva Klintström

**Affiliations:** 1 Department of Biomedical Engineering and Health Systems, KTH Royal Institute of Technology, Huddinge, Sweden; 2 Division of Diagnostics and Specialist Medicine, Department of Health, Medicine and Caring Sciences, Linköping University, Linköping, Sweden; 3 Department of Acute Internal Medicine and Geriatrics, Linköping University Hospital, Linköping, Sweden; 4 Center for Medical Image Science and Visualization, CMIV, Linköping, Sweden; 5 Department of Health, Medicine and Caring Sciences, Linköping University, Linköping, Sweden; 6 Institute of Lightweight Design and Structural Biomechanics, TU Wien, Gumpendorfer Straße 7, Vienna, 1060 Austria; 7 Department of Radiology in Linköping and Department of Health, Medicine and Caring Sciences, Division of Diagnostics and Specialist Medicine, Linköping University, Linköping, Sweden

**Keywords:** CBCT, DXA, Bone structure, Segmentation, Structure analysis, Osteoporosis, Bone imaging, Computed tomography

## Abstract

Fracture liaison services are essential to mitigate underdiagnosis and undertreatment of osteoporosis-related fractures. However, it often suffers from limited access to dual-energy X-ray absorptiometry (DXA) or high-resolution peripheral quantitative CT equipment. This in vivo study of 21 patients aims to evaluate the feasibility of dental cone beam CT (dCBCT) to analyse bone properties of human wrists, comparing with DXA and finite element (FE) analysis. dCBCT grey-scale values were transformed to HU using a phantom containing materials with known HU values. Strong correlations were found between bone mineral content (BMC) from dCBCT and DXA (*r* = 0.78 to 0.84, *p* < 0.001), as well as between BMC from dCBCT FE-predicted stiffness (*r* = 0.91) and maximum force (*r* = 0.93), *p* < 0.001. BMC values from dCBCT were higher than DXA measurements (2.34 g vs. 1.5 g, *p* < 0.001). Cortical thickness strongly correlated to bone mineral density (BMD) from dCBCT (*r* = 0.83, *p* < 0.001). No statistically significant correlations were found between trabecular bone microstructure and FE predictions. The results indicate the feasibility to analyse osteoporosis related bone properties of human wrists from corrected dCBCT data. The dCBCT values of BMD and BMC were strongly correlated with DXA.

## Introduction

Osteoporosis-related fractures, of e.g., the wrist, hip and vertebrae are common and recurrent and often interfere with the ability to perform daily activities^1,2^. These fractures result in high healthcare costs and great suffering for the affected individuals^3^. North European countries like Sweden and Denmark have an incidence of > 500 hip fractures per 100,000 individuals annually, affecting every second woman and every fourth man after the age of 50^4^. The condition is extensively undertreated^5^. An important step to improve the underdiagnosis and undertreatment after an osteoporosis-related fracture is by implementing fracture liaison services (FLS) to secure a coordinated handling, including diagnostic assessment and treatment^6^. The FLS is normally coordinator-based and aims to identify and correctly investigate and treat all women and men over the age of 50 suffering an osteoporosis-related fracture^6^.

Today’s reference method for evaluating osteoporosis is dual-energy X-ray absorptiometry (DXA). This method was introduced over three decades ago^7^ and is widely used today^8^. DXA can measure areal bone mineral density (aBMD) in the central and peripheral skeleton. Another established method for osteoporosis detection is high-resolution peripheral quantitative computed tomography (HR-pQCT), which can analyse volume BMD (vBMD) and the cortical and trabecular bone structure in the wrist and ankle^9,10^. A drawback is that examinations with both these methods often require an extra visit and risk delayed diagnosis. Another drawback with HR-pQCT is the limited number of these devices available worldwide.

Dental cone beam CT (dCBCT) is a diagnostic high-resolution 3D-CT device for imaging the maxillo-facial region at isotropic voxels^11^. With some adjustments, these devices could be used to visualise the peripheral skeleton^12^. In vitro data from dCBCT has shown strong correlations to micro-CT data regarding bone microstructure^13,14^. A drawback is that dCBCT devices do not provide Hounsfield units (HU). The provided grey-scale values are, by some studies, suggested to be reliable^15^, yet other studies show that these values differ between devices and between different fields of view (FOV)^16^. This behaviour implies a drawback of this method for analysing vBMD. The CBCT data could be corrected by using the relative attenuation ratio between materials in the imaged FOV. Studies have shown that such calibrations are feasible^17^.

Besides bone structure and mineral content, mechanical properties like bone strength and stiffness are important for resilience to fractures. Such mechanical properties can be analysed by finite element (FE) methods from DXA and CT data^18,19^. Studies indicate that dCBCT data can be used to assess microstructural and mechanical parameters of fresh-frozen wrist specimens^20^.

If dCBCT data, including high-quality data on microarchitecture, could be used for bone property analysis, the technique would improve osteoporosis care.

### Aim

This in vivo study aims to evaluate the feasibility of analysing bone properties related to osteoporosis in human wrists by automatic 3D bone structure analysis of corrected dental CBCT data.

## Methods

### Study design

The present study was a cross-sectional observational study comparing dCBCT to DXA results.

### Study participants

The participants in this study were recruited in 2019 from the osteoporosis unit, department of Endocrinology at Linköping University Hospital, where they were referred for bone mineral analysis by DXA. The inclusion criteria were: (1) women and men, (2) age ≥ 18 years, and (3) with an ability to understand the information of the study. Written informed consent was obtained from each participant before being included in the study.

### Methods

All patients underwent DXA examinations of the left forearm using a Horizon A system (SN/200032) (Hologic Inc., Bedford. MA, USA). BMD and BMC measurements from the upper distal radius (UD) were used in further analysis.

**Figure 1:**
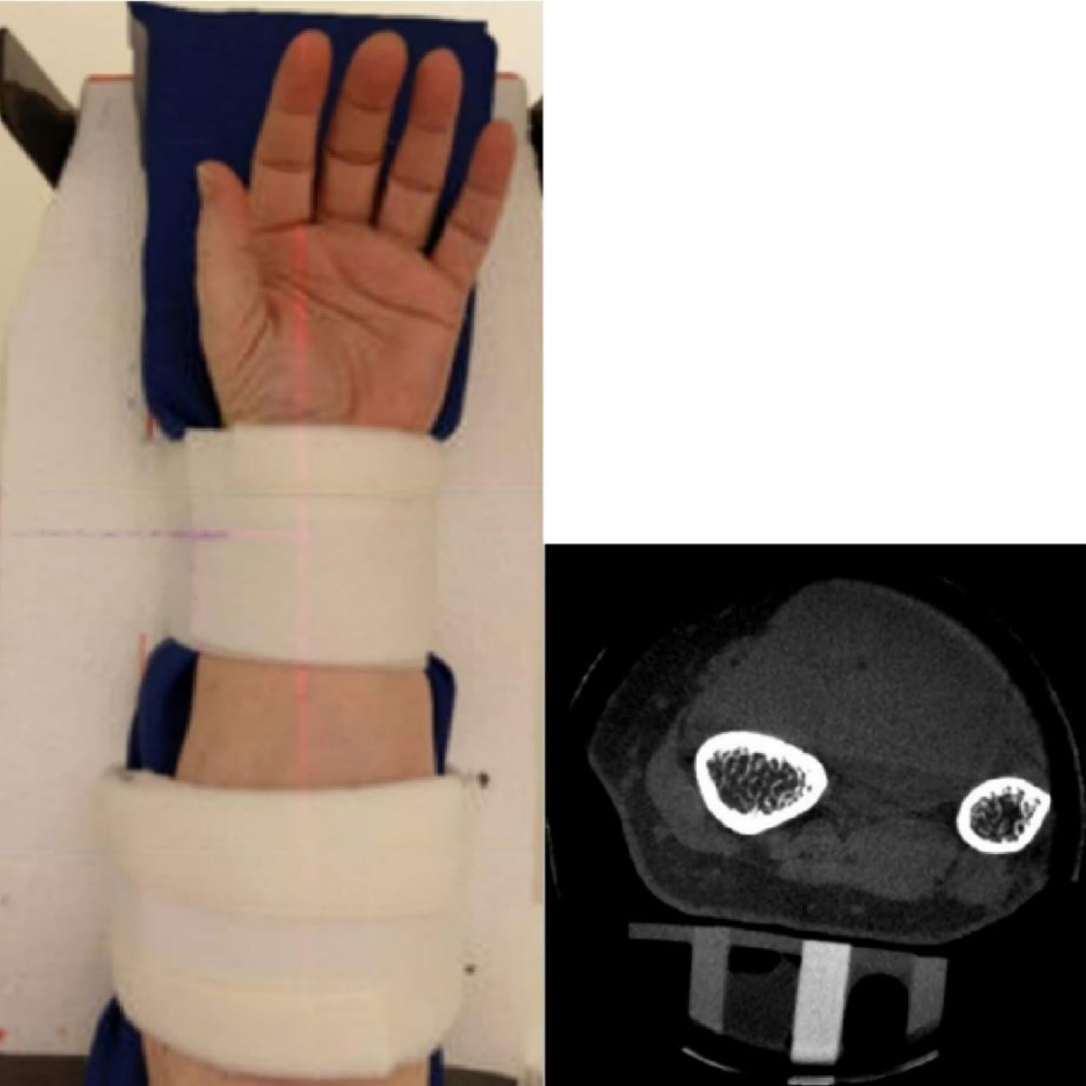
Photo of the custom-made holder for fixation of the wrist at imaging in dCBCT and one axial slice at the location of the phantom, including air, two pieces of polyoxymethylene and one piece of polytetrafluoroethylene.

The dCBCT examinations of the left forearm were performed using a 3D Accuitomo 80 (J. Morita MFG., Kyoto, Japan) with a spatial resolution better than 2 lp/mm at 10% MTF. During imaging, the patients were positioned supine on a height-adjustable hospital bed, with their left arm stabilised in a custom-made holder that incorporated a phantom embedded with materials of known HU; Polyoxymethylene (POM): HU of 300, Density: 1.41 g/cm^3^ Hydroxyapatite (HA) and Polytetrafluoroethylene (PTFE)/ TEFLON, HU of 950, Density: 2.2 g/cm^3^ HA (placed centrally) (Fig. 1).

The imaging parameters for dCBCT were a tube current of 5 mA, a tube voltage of 85 kV, and a FOV of 60 mm, resulting in 125 μm isotropic voxels.

The analysed dCBCT volumes had lengths of 10 mm starting 9.5 mm from a reference line placed at the endplate of the distal radius, as recommended for HR-pQCT imaging^21^. The dCBCT volumes were in the same positions as the areas imaged by DXA. Before the bone analyses, the dCBCT volumes were aligned according to the long axis of the lower arm using an in-house developed MATLAB code MATLAB version R2022a (Mathworks, Natick, MA, USA). Before segmentation, the reconstructed grey values were transformed into HU values using scan-specific calibration curves. Each calibration curve was obtained by fitting a linear function to phantom data at three points corresponding to air, polyoxymethylene and polytetrafluoroethylene. The grey values were taken using manually defined ROIs, and Hounsfield values were calculated using tabulated cross-section data at 48 keV corresponding to the effective energy of an 85 kV spectrum. Tabulated cross-section data were also used to derive a relation between HU values and the mass density of calcium in g/cm³ at 48 keV. Reconstructed grey values converted to the mass density of calcium were used to determine vBMC and vBMD for the whole volume (cortical, transitional, and trabecular) and the cortical and trabecular parts, respectively.

Multiple instances of the fast-level-set implementation in MiaLab (mialab.org) were used to segment the bone from the background and separate the cortical zone from the transitional and trabecular zones^22^.

An in-house-developed version of automated region-growing (ARG) segmentation code using MATLAB version R2022a (Mathworks, Natick, MA, USA) was used to segment the trabecular bone. The ARG-segmentation code uses an iteratively looser homogeneity threshold to segment bone from background voxels. It then selects the iteration with the lowest combined inhomogeneity of bone and background as the optimal segmentation. The code was used in previous studies from our group and is explained in more detail in^23^.

After the alignment in MATLAB and localisation of the endplate of the distal radius, no manual intervention was needed. The delineation of cortical, transitional, and trabecular parts and all analyses of trabecular bone microstructure parameters using the ARG segmentation code were thereafter fully automatic, taking approximately 8 min for each wrist.

Biomechanical properties were derived by FE methods on the segmented and corrected dCBCT data after a cortical porosity segmentation using a threshold of 600 mg/cm^3^ HA. Micro-FE models were created by conversion of the image voxels into linear hexahedral finite elements with a side length of 125 μm using Medtool 4.5 (Dr. Pahr Ingenieurs e.U., Pfaffstätten, Austria). The elements were assigned isotropic, linear elastic material properties with a Young’s modulus of 15 GPa and a Poisson’s ratio of 0.3^24^. All nodes at the proximal cross-section were fully constrained, and an axial displacement of 0.1 mm (approximately 1% axial strain) was imposed on the distal cross-section to simulate axial compression. Lateral degrees of freedom at the distal cross-section were left unconstrained. All models were solved using ParOSol^25^. Stiffness was evaluated as the axial force divided by the axial displacement, and the maximum force was computed by scaling the results such that the Pistoia criterion (critical effective strain of 0.7% and a critical fraction of overstrained elements of 2%^24^ was met. Strength was computed by dividing the maximum force by the mean cross-sectional area of the bone. Note that all computed quantities were independent of the chosen displacement magnitude of 0.1 mm, as the FE model was entirely linear, and the maximum force was determined by scaling the results.

### Statistical methods

Data were compared using Pearson correlation with 95% confidence intervals, linear regression using *R*^*2*^ values, and the Bland Altman test in MATLAB version 2023b. For comparisons of means, the Mann-Whitney U-test was used. To test for normality, the Shapiro-Wilk test was used. Graphs and figures were created using MATLAB and InDesign.

### Ethics

The study was ethically approved by the regional ethical committee in Linköping, Sweden, and the local radiation committee (2015/474 − 31) and performed in accordance with all relevant guidelines and regulations.

## Results

### Study population characteristics

**Figure 2:**
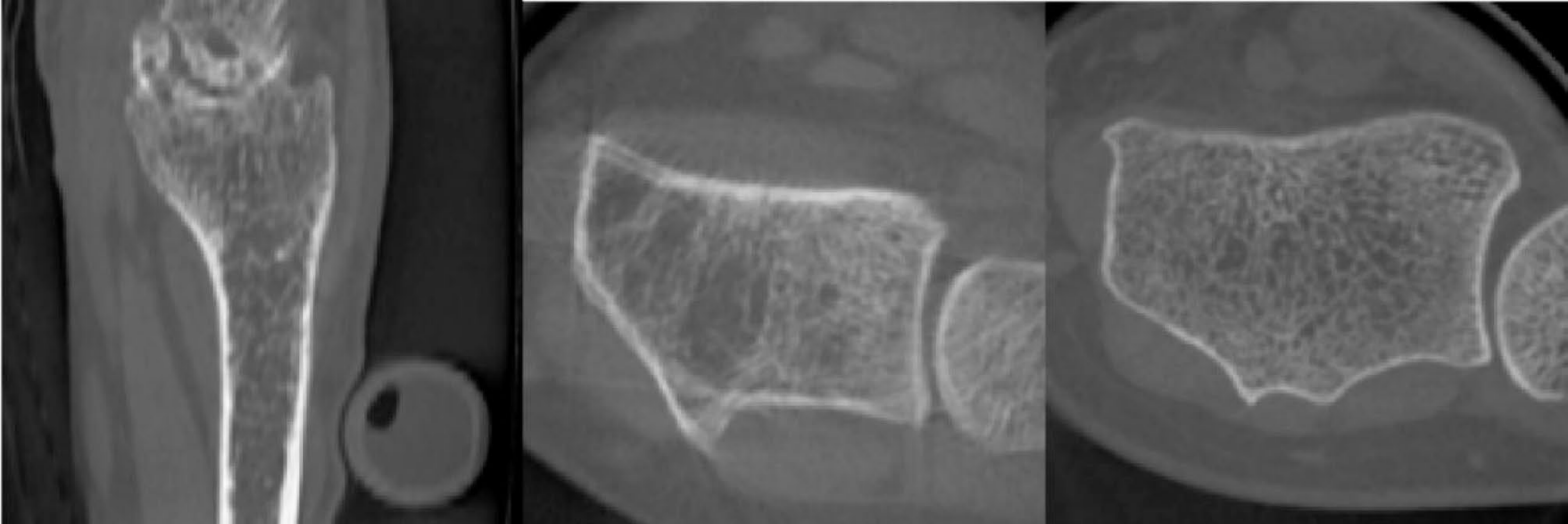
To the left a sagittal CBCT slice of the wrist excluded due to complex anatomy. In the middle an axial slice from one of the patients excluded due to movement artifacts and to the right an axial slice demonstrating a patient included in the study.

In total, 26 patients were recruited for the study. Of those, five patients were excluded, three due to movement artifacts in the dCBCT, one due to the field of view (FOV) not including the calibration phantom, and one due to complex wrist anatomy making identification of landmarks impossible (Fig. 2). This resulted in 21 individuals included in the analysis. Of these, 14 were females and 7 males. Mean age 67 years (median 67, range 42–83), mean BMI 26 + 3 kg/m^2^.

A history of previous osteoporotic fracture was reported in 38% of patients. One-fourth (24%) were on osteoporotic treatment, including bisphosphonates (*n* = 3) and Denosumab (*n* = 2). Regarding risk factors for osteoporosis, 29% reported heredity of osteoporosis, 24% reported cortisone use, 5% rheumatoid arthritis, 33% hyperparathyroidism (present or former), and 33% smoking (present or former).

### Bone measurements in dCBCT and DXA

**Figure 3:**
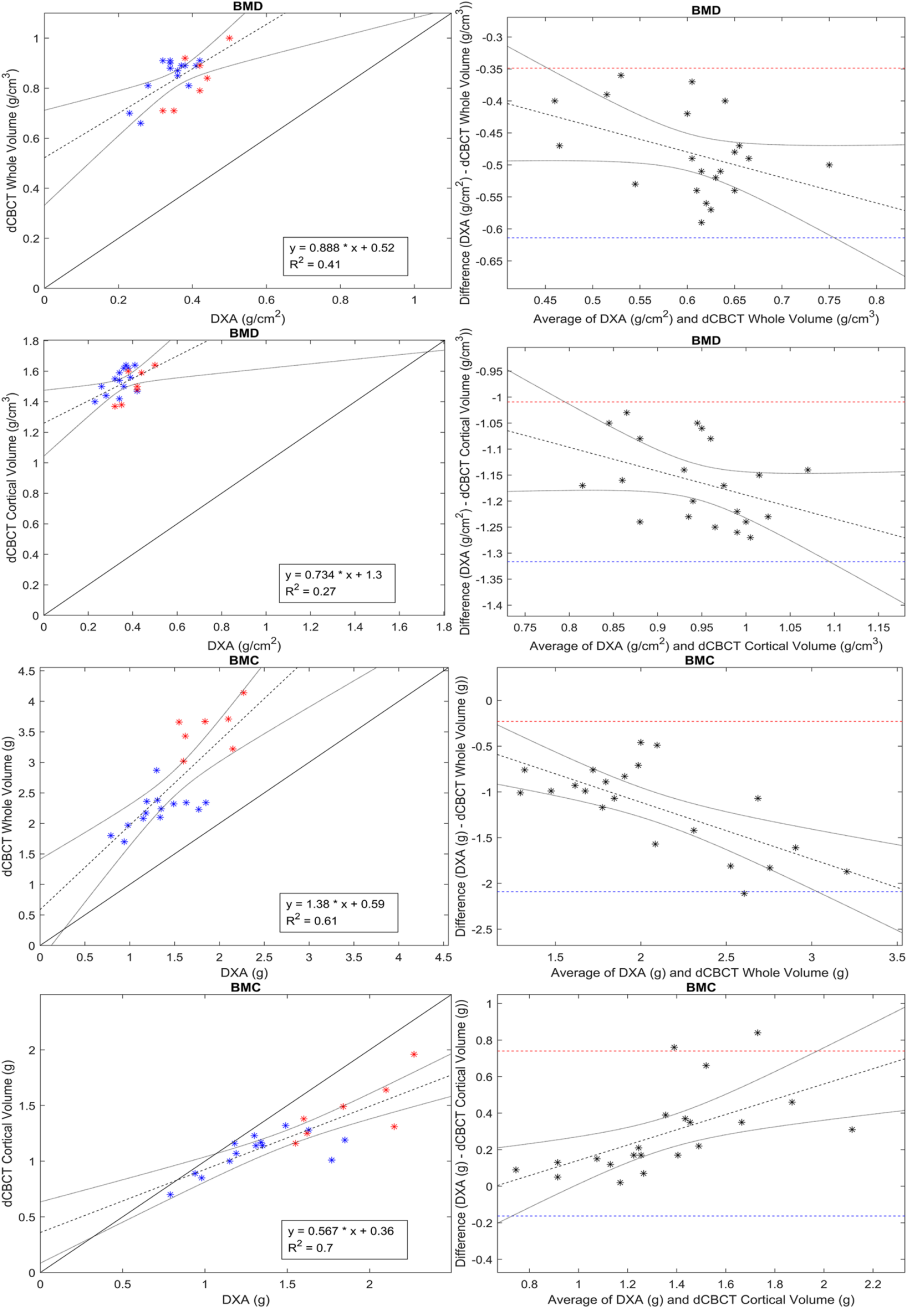
Linear regression r^2^ to the left and Bland Altman plots to the right demonstrate relations between BMD and BMC from DXA with those derived from calibrated dCBCT data. Blue dots represent women, and red represents men.

This study found strong correlations between BMC derived from the corrected dCBCT data and BMC derived by DXA and moderate to strong correlations for BMD of the human wrists examined in vivo (Table 1; Fig. 3).This was regardless of DXA measuring aBMD in g/cm^2^ while dCBCT measures vBMD in g/cm^3^.

**Table 1:** Pearson correlation coefficients for the bone Mineral Content and Bone Mineral Density (with a large D) measurements.

		dCBCT			
		BMD (g/cm^3^)		BMC (g)	
		Whole Bone	Cortical	Whole Bone	Cortical
DXA	BMD (g/cm^2^)	*r* = 0.64 (0.28 : 0.84) *p* = 0.002	*r* = 0,52 (0.11 : 0.77) *p* = 0.017		
	BMC (g)			*r* = 0.78 (0.52 : 0.90) *p* = 0.034	*r* = 0.84 (0.63 : 0.93) *p* < 0.001

*CBCT* dental cone beam computed tomography, *DXA* Dual-Energy Absorptiometry

*BMD* bone mineral density, *BMC* bone mineral content

Measurements of BMC from the whole wrist bone volume were significantly higher for dCBCT than DXA (median of all subjects of 2.34 g for dCBCT vs. mean of 1.50 g for DXA, *p* < 0.001), showing an overestimation by dCBCT compared to DXA (Table 2; Fig. 3).

**Table 2:** Descriptive statistics.

	Female	Male	Total
BMD DXA (g/cm^2^)	0.34 ± 0.06	0.40 ± 0.06	0.36 ± 0.06
BMD dCBCT Whole bone (g/cm^3^)	0.89 [0.84 ; 0.88]	0.84 [0.73 ; 0.91]	0.88 [0.81 ; 0.90]
BMD dCBCT Cortical (g/cm^3^)	1.54 ± 0.08	1.51 ± 0.11	1.53 ± 0.09
BMC DXA (g)	1.31 ± 0.31	1.88 ± 0.30	1.50 ± 0.40
BMC dCBCT whole bone (g)	2.24 [2.08 ; 2.34]	3.66 [3.27 ; 3.70]	2.34 [2.15 ; 3.27]
BMC dCBCT cortical (g)	1.08 ± 0.18	1.46 ± 0.27	1.21 ± 0.27
C.Th (mm)	1.25 [1.25 ; 1.32]	1.30 [1.09 ; 1.34]	1.25 [1.19 ; 1.32]
Trabecular BV/TV (%)	44 ± 5	43 ± 1	44 ± 4
Tb.Th (mm)	0.63 ± 0.06	0.62 ± 0.04	0.63 ± 0.06
Tb.Sc (mm)	1.29 ± 0.33	1.19 ± 0.08	1.26 ± 0.27
Tb.Nd (1/mm^3^)	0.99 ± 0.24	1.07 ± 0.13	1.02 ± 0.21
TV (mm^3^)	2 580 [2 462 ; 2 706]	4 416 [3 751 ; 4 792]	2 706 [2 505 ; 3 751]
Mean area (mm^2^)	255 [243 ; 267]	436 [370 ; 473]	267 [247 ; 370]
Stiffness (N/mm)	169 783 ± 24 526	240 613 ± 32 974	193 393 ± 43 456
Strength (N/mm^2^)	33 ± 4.7	29 ± 4.0	32 ± 4.9
FMAX (N)	8 561 ± 1 107	12 153 ± 1 509	9 758 ± 2 119
TBV (mm^3^)	1 561 [1450 ; 1623]	2 499 [2 170 ; 2 605]	1 623 [1 495 ; 2 170]
BV/ TV Whole Volume (%)	60 ± 6	56 ± 4	59 ± 5

Mean values +/- standard deviations or median [first quartile ; third quartile] depending on whether the parameter is normally distributed according to the Shapiro-Wilk test

*BMD* bone mineral density, *DXA* dual energy absorptiometry, *CBCT* cone beam computed tomography, *Whole bone volume* cortical + transitional + trabecular parts, *BMC* bone mineral content, *C.Th* cortical thickness, *BV/TV* bone volume over total volume, *Tb.Th* trabecular thickness, *Tb.Sc* trabecular spacing, *Tb.Nd* trabecular nodes, *TV* total volume, *FMAX* maximum force, *TBV* total bone volume

### FE analysis vs. dCBCT and DXA bone mineral measurements

Stiffness, strength, and maximum force, derived by FE analysis from the calibrated dCBCT data, were compared to BMD and BMC (Tables 2 and 3). Strong correlations were found between stiffness (*r* = 0.91), maximum force (*r* = 0.93), and BMC calculated from dCBCT-data as well as between strength and BMD calculated from dCBCT-data (*r* = 0.68). Comparing BMC measurements from DXA with the FE analyses resulted in a correlation of 0.69 for stiffness and 0.71 for maximum force. Weak correlations and no statistical significance were demonstrated for strength compared to DXA measurements. More information is in Table 3.

**Table 3:** Linear regression comparing FE analysis to DXA and dCBCT-derived bone mineral parameters.

	FEM		
	Stiffness	Strength	Maximum Force
dCBCT BMD	*r* = 0.17 (-0.29 : 0.56) *p* = 0.472	*r* = 0.68 (0.36 : 0.86) *p* < 0.001	*r* = 0.19 (-0.27 : 0.57) *p* = 0.42
dCBCT BMC	*r* = 0.91 (0.78 : 0.96) *p* < 0.001	*r* = -0.33 (-0.67 : 0.12) *p* = 0.147	*r* = 0.93 (0.84 : 0.97) *p* < 0.001
DXA BMD	*r* = 0.6 (0.23 : 0.82) *p* = 0.00376	*r* = 0.2 (-0.25 : 0.58) *p* = 0.385	*r* = 0.63 (0.27 : 0.83) *p* = 0.00225
DXA BMC	*r* = 0.69 (0.37 : 0.86) *p* < 0.001	*r* = -0.12 (-0.52 : 0.33) *p* = 0.616	*r* = 0.71 (0.4 : 0.87) *p* < 0.001

*dCBCT* dental cone beam computed tomography, *DXA* dual energy absorptiometry, *BMD* bone mineral density, *BMC* bone mineral content. Stiffness, Strength, and Maximum Force were derived with Finite element (FE) analysis

### FE analysis vs. bone microstructure parameters derived by dCBCT

Cortical thickness showed strong correlations to BMD from the dCBCT data (*r* = 0.83) and had correlations of *r* = 0.44, *r* = 0.45 and 0.5 to the FE calculations (Table 4). The total bone volume strongly correlated to BMC derived from dCBCT data at *r* = 0.91 and stiffness and maximum force. BV/TV was rather strongly related to strength. Regarding other trabecular bone microstructure parameters (Tb.Th, Tb.Sc and Tb.Nd) and their correlations to bone properties, only Tb.Sc and Tb.Nd demonstrated p-values lower than 0.05, which was only demonstrated for BMD measurements of dCBCT data.

**Table 4:** Linear regression comparing FE analysis to bone microstructure parameters.

	dCBCT		FEA		
	BMD	BMC	Stiffness	Strength	Maximum Force
BVTV	*r* = 0.47 (0.042 : 0.75) *p* = 0.034	*r* = -0.032 (-0.46 : 0.41) *p* = 0.892	*r* = 0.2 (-0.26 : 0.58) *p* = 0.390	*r* = 0.7 (0.38 : 0.87) *p* < 0.001	*r* = 0.19 (-0.26 : 0.58) *p* = 0.407
Tb.Th	*r* = -0.4 (-0.71 : 0.036) *p* = 0.071	*r* = -0.18 (-0.57 : 0.27) *p* = 0.427	*r* = 0.009 (-0.42 : 0.44) *p* = 0.969	*r* = 0.027 (-0.41 : 0.45) *p* = 0.908	*r* = -0.044 (-0.47 : 0.4) *p* = 0.850
Tb.Sc	*r* = -0.56 (-0.8 : -0.17) *p* = 0.008	*r* = -0.3 (-0.65 : 0.15) *p* = 0.189	*r* = -0.25 (-0.62 : 0.2) *p* = 0.270	*r* = -0.28 (-0.64 : 0.17) *p* = 0.217	*r* = -0.3 (-0.65 : 0.15) *p* = 0.187
Tb.Nd	*r* = 0.56 (0.17 : 0.8) *p* = 0.008	*r* = 0.25 (-0.2 : 0.62) *p* = 0.267	*r* = 0.11 (-0.33 : 0.52) *p* = 0.621	*r* = 0.17 (-0.29 : 0.56) *p* = 0.469	*r* = 0.18 (-0.28 : 0.56) *p* = 0.446
C.Th	*r* = 0.83 (0.63 : 0.93) *p* < 0.001	*r* = 0.4 (-0.044 : 0.71) *p* = 0.076	*r* = 0.44 (0.013 : 0.73) *p* = 0.045	*r* = 0.5 (0.091 : 0.77) *p* = 0.020	*r* = 0.45 (0.025 : 0.74) *p* = 0.040
Total Bone Volume	*r* = -0.27 (-0.63 : 0.18) *p* = 0.235	*r* = 0.91 (0.79 : 0.96) *p* < 0.001	*r* = 0.81 (0.59 : 0.92) *p* < 0.001	*r* = -0.32 (-0.66 : 0.13) *p* = 0.158	*r* = 0.83 (0.62 : 0.93) *p* < 0.001

*dCBCT* dental cone beam computed tomography, *FEM* finite element methods

*BMD* bone mineral density, *BMC* bone mineral content, *BV/TV* bone volume over total volume, *Tb.Th* trabecular thickness, *Tb.Sc* trabecular spacing, *Tb.Nd* trabecular nodes, *C.Th* cortical thickness

## Discussion

In this study, we have demonstrated the ability to image human wrists in vivo using a dental cone beam CT (dCBCT) device and, from those data, calculate bone mineral density (BMD) and bone mineral content (BMC), which were strongly correlated to DXA-measurements.

At dCBCT imaging, the wrists were placed in a custom-made holder, including materials with known HU values, to enable scan-specific calibration. The corrected data sets were segmented using a code based on fast level sets and automated region growing (ARG)^26,27^. Stiffness and maximum force derived by finite element (FE) analysis showed correlations to DXA measurements, with r varying from 0.60 to 0.71. According to the study from Akoglu, such correlations could be considered moderate to high^28^. Correlations between cortical thickness and BMD were strong, while the correlations between cortical thickness and mechanical properties were moderate.

In a study of 126 trabecular bone samples examined by micro-CT, BV/TV was the best single determinant of stiffness and strength^29^. This agrees with the result of the study where BV/TV was strongly and significantly correlated with strength. The use of structural stiffness instead of elastic and shear moduli may explain why no correlation between BV/TV and stiffness was seen in our study. Besides BV/TV, the trabecular bone microstructure showed very low and no significant correlation to the mechanical properties of the imaged part of the wrist. This disagrees with the study from Carbonare and Giannini, where bone microstructure was shown to be an important determinant of bone strength^30^. In that study, bone microstructure was studied two-dimensionally by microscope. In another ex-vivo study, a combination of BMD and trabecular structure better predicted the biomechanical properties than either of these parameters alone^31^. Our study is in vivo, causing risk for motion artefacts, and is conducted at a lower resolution, 0.125 mm voxels, which may result in overestimation of, e.g., trabecular thickness (Tb.Th) and underestimation of trabecular nodes (Tb.Nd). In a previous ex vivo study from our research group on small, defatted wrist bone specimens using the same device but at a higher resolution (0.08 mm), Tb.Th had a mean of 0.48 mm and Tb.Nd a mean of 1.48 (1/mm^3^)^23^. In our new in-vivo study, the mean Tb.Th was 0.65 mm and mean Tb.Nd was 1.02, possibly demonstrating such over- and underestimations. Those specimens were defatted and taken from a different cohort, making direct comparisons impossible. Even minor patient motions during the 17 s imaging may explain disagreements between this in-vivo study and previous ex-vivo studies regarding bone microstructure.

Regarding mechanical properties derived by FE analyses in the wrists in vivo, there are a few studies^32–34^, but to our knowledge, all those studies are on HR-pQCT data. In an ex vivo study of 19 wrist specimens from individuals at a mean age of 68 years, scanned by dCBCT at a resolution of 0.75 mm (reconstructed to 0.60 mm), measurements of stiffness and maximum force derived by FE analyses showed very strong correlations to micro-CT^20^. The mean stiffness values at 175 kN/mm and maximum force at 8 646 N are in the same range as those from our in vivo study of 21 human wrists from individuals at a mean age of 67 years. In our study, the mean stiffness was 193 kN/mm, and the mean maximum force was 9758 N, indicating that our measurements seem reasonable.

Our study’s main concern is the conversion accuracy from CBCT grey levels to HU values. The usability of a linear relation was demonstrated in ref^35^. Nevertheless, it is known that the grey values differ in the imaged FOV^36^. Our phantom, including material with known HU values, used for the calibration was placed in the periphery of the imaged FOV. That may have impacted the segmentation of the more centrally located trabecular bone structure. In practice, a CBCT scanner user must determine uncertainties associated with the calibration curve.

In our study, the segmented cortical volumes were strongly and significantly correlated to the calculated BMD but showed moderate correlations to the derived mechanical properties. A combination of cortical thickness and microstructural variations may impact the correlations to mechanical properties. Due to a limited number of studied individuals, such multivariate analysis was not performed, but would be interesting to examine in a larger study population.

Studies have shown that HR-pQCT examinations of the distal extremities, preferably the wrist, may predict major osteoporotic fractures, such as of the hip^37^. Considering the limited number of HR-pQCT devices today, only a limited minority of patients are scanned, primarily for research. Increased access to these kinds of analyses, including bone microstructure, could impact osteoporosis care in the future. Since dental CBCT devices are more frequently available in public and private healthcare, we chose this for our study. The dental CBCT device used is for patients in the sitting position and requires adaptation before imaging. Devices for imaging in the lying position might facilitate the examination of the often old patients of the relevant population^12^. CBCT devices for extremity imaging are available and may be helpful for osteoporosis imaging if the resolution is sufficient^38,39^. Many in-vitro and a few in-vivo studies have demonstrated good results for bone microstructure and other bone properties like FEA using dCBCT with micro-CT as a reference standard^14,20,40^. Our research group studied bone properties by analysing data from several different clinical CT devices via ARG-based code. Strong correlations to micro-CT were demonstrated for small bone^27,38,40^ and whole wrist specimens^41^.

### Limitations and considerations

The low number of patients included in the present study is a limitation.

A consideration is the relatively long scanning time from the used dCBCT, 17 s, that may result in motion artefacts compromising bone structure analyses. Therefore, clear instructions to the patients before and accurate fixation during scanning are crucial. This study used a device for patients in a sitting position and a custom-made wrist holder, which may not be ideal for imaging and analyses. Such obstacles can be overcome in the future either by manufacturer-provided holders or by imaging patients in the lying down position.

The overestimation of BMC by dCBCT when compared to DXA is a concern and a limitation for comparisons between the two modalities. One possible explanation for this overestimation could be if the due to beam hardening correction applied by the manufacturer if they result in capping. This could then result in higher greyscale-values in the centre of the scanned volume. This would be a problem in this study since the phantom is placed at the edge of the scanned FOV while the analysed wrist is placed close to the centre.

In addition, only linear FEA was used in this study. Maximum force predictions of linear FEA were shown to correlate well with in vitro experimental measurements, but may still show a quantitative mismatch^42^. Also segmentation errors can contribute to a quantitative mismatch in the FEA predictions. However, correlations are expected to be less affected by these segmentation errors^43^. In the future, FE models based on segmented images could also be compared to FE models that avoid segmentation and implement density-based material laws^44^.

## Conclusion

The results from this in vivo study of 21 individuals indicated that it was feasible to analyse bone properties of human wrists related to osteoporosis from calibrated dental Cone Beam CT (dCBCT) data. The dCBCT received values of BMD and BMC were strongly correlated with the DXA measurements. Another interesting finding was the strong and significant correlation between BV/TV and strength measures derived by FEA.

## Data Availability

Pseudonymised data may be made availible upon reasonable request made to the corresponding author.
